# Chemical accuracy in QM/MM calculations on enzyme-catalysed reactions

**DOI:** 10.1186/1752-153X-1-19

**Published:** 2007-07-05

**Authors:** Adrian J Mulholland

**Affiliations:** 1 Centre for Computational Chemistry, University of Bristol, Bristol BS8 1TS, UK

## Abstract

Combined quantum mechanics/molecular mechanics (QM/MM) modelling has the potential to answer fundamental questions about enzyme mechanisms and catalysis. Calculations using QM/MM methods can now predict barriers for enzyme-catalysed reactions with unprecedented, near chemical accuracy, i.e. to within 1 kcal/mol in the best cases. Quantitative predictions from first-principles calculations were only previously possible for very small molecules. At this level, quantitative, reliable predictions can be made about the mechanisms of enzyme-catalysed reactions. This development signals a new era of computational biochemistry.

## Background

Ever since the catalytic power of enzymes was first recognised, chemists have wondered and argued about how they work. Enzymes are outstandingly efficient natural catalysts. Better understanding of the mechanisms by which they achieve these catalytic properties promises technological spin-offs such as routes to new drugs (many drugs are enzyme inhibitors, which bind to enzymes and prevent them from functioning), analysis of the effects of genetic variation and mutation (for example in predicting individual metabolism of pharmaceuticals); and the design of new catalysts (for example biomimetic catalysts or engineered enzymes). There is great interest in developing protein catalysts for practical applications, for instance in the pharmaceutical, chemical and biotechnology industries. Computational modelling has a vital role to play in these developments: unstable species such as transition states and reaction intermediates are crucial to questions of reactivity, but cannot be studied directly by experiment in systems as complex as enzymes. The field of enzyme reaction modelling has grown enormously in recent years and has matured to the point that computational enzymology is increasingly recognised as essential for understanding these fascinating biological catalysts [1-4]. Recent calculations [5] bring a new level of accuracy to bear on the problem, essential for quantitative conclusions and comparisons with experiment.

Combined quantum mechanics/molecular mechanics (QM/MM) methods allow enzyme reactions to be modelled: a small region at the active site (where the reaction happens) is treated by a quantum mechanical electronic structure method; this region interacts with the protein and solvent environment, which are included more simply (though in atomic detail) by an empirical 'molecular mechanics' force field [1,2,4,6]. This approach combines the simplicity and speed of the MM treatment of the protein structure with the flexibility and power of a quantum chemical treatment (which allows modelling of bond breaking and making, and electronic polarization). Until recently, QM/MM investigations of enzymes have generally been limited to relatively low levels of QM theory, such as semi-empirical methods or density functional theory (DFT). Semi-empirical methods are computationally cheap, fast enough for QM/MM molecular dynamics simulations, but error-prone, and give reaction energies and barriers that can be in error by 10 kcal/mol or more. DFT (especially with the B3LYP hybrid functional) offers improved accuracy, and has opened new classes of enzymes (particularly metalloenzymes) to computational investigation [7], such as studies of cytochrome P450 enzymes that metabolize drugs in the body [8,9]. These DFT methods, however, lack key physical interactions, such as dispersion, which are important in the binding of ligands to proteins. DFT often gives barrier heights that are too low by several kcal/mol, and it does not offer a route to their systematic improvement or testing, making it difficult to assess the accuracy of results. Other modelling methods such as the empirical valence bond technique can give excellent results for enzyme activation energies [3,10], and have provided important insights into the basic causes of catalysis. Such empirical approaches, however, require extensive fitting to experimental data, and do not consider the electronic structure explicitly.

Enzymology has been marked by vigorous debates and controversial proposals about enzyme mechanisms, and about the physical origins of enzyme catalysis. Identifying the chemical mechanisms of enzymes has proved difficult: it is often hard to differentiate between alternative proposals, and many 'textbook' mechanisms are probably incorrect in important details. Recent controversies over enzyme catalysis include proposals of 'low-barrier' hydrogen bonds [11-14], 'near-attack conformations' [4,15,16], the role of enzyme dynamics in catalysis [2,3], quantum tunnelling [17] and entropic effects [3]. The applicability of transition state theory to enzyme reactions has been questioned. These arguments have often proved extremely difficult to resolve, because the complexity and large size of enzymes makes experimental analysis very difficult. Atomistic simulations have a potentially vital role to play in these debates, in the interpretation of experimental data, and in providing a molecular level picture of reactions in enzymes. Calculations have the potential to identify probable mechanisms, and to analyse key interactions and catalytic effects. For quantitative comparisons with experiments, and reliable predictions, high-level electronic structure methods are needed. Recent work by Claeyssens *et al*. has shown that it is now possible to achieve an unprecedented level of accuracy for enzyme-catalysed reactions in QM/MM calculations [5]. Calculated activation energies for two enzyme reactions agree very well with experiment; indeed the agreement is so good that, given the known properties of the high-level methods now available, it is clear that near chemical accuracy (1 kcal/mol) can be achieved in calculations on enzyme-catalysed reactions. Such quantitative predictions in first principles calculations were only previously possible for very small molecules. These findings herald a new era of computational biochemistry.

## Discussion

The calculations focused on two enzymes that have become paradigms for computational investigations: chorismate mutase (CM) and *para*-hydroxybenzoate hydroxylase (PHBH). Both have been studied previously with lower-level (semi-empirical and ab initio) QM/MM methods [4,15,18-23], and a wealth of experimental data is available for comparison. CM catalyses the Claisen rearrangement of chorismate to prephenate, a key step in the biosynthesis of essential aromatic amino acids. The reaction catalysed by PHBH, an electrophilic aromatic substitution involving a hydroperoxyflavin cofactor, is important in the microbial breakdown of aromatic pollutants and lignin from wood. Earlier modelling had been encouraging, for example in showing correlations between experimental rates and calculated activation energies for the key step in PHBH [24], in addition to identifying groups involved in lowering the energy barrier to reaction by transition state stabilization in both enzymes [4,22,23]. The barriers calculated at these lower levels of QM/MM treatment were, however, typically significantly different from experiment by 50% or more.

Computational chemistry is notorious for its love of acronyms, which can make judging the results of calculations difficult for non-specialists. The 'gold standard' of quantum chemistry is provided by first principles – '*ab initio*' – methods that include correlation between electrons. They allow the calculation of rate constants for gas-phase reactions involving very few atoms with an accuracy similar to that obtained experimentally [25]. For example, the hierarchy of *ab initio *electron correlation methods MP2 (Møller-Plesset second-order perturbation theory), CCSD (coupled-cluster theory with single and double excitations) and CCSD(T) (CCSD with a perturbative treatment of triple excitations) provide a a route to converging reliably to high accuracy. These methods have previously been limited to small molecules because of their very large computational expense, which increases enormously with the size of the system studied. This large increase in computational cost is mostly due to the fact that the molecular orbitals are delocalized over the whole system. Physically, dynamic correlation between electrons is a short-ranged phenomenon in covalent molecules. The vital electron correlation effects can therefore be treated accurately and efficiently by localizing the molecular orbitals. Using a range of approximations it is possible to achieve effective linear scaling of the computational requirements of calculations with molecule size. With recent developments[26] it is now possible to treat systems of the size of typical QM regions in QM/MM calculations on enzymes (for example 24 atoms in CM; 49 atoms in PHBH).

The model of PHBH, constructed by Thiel *et al*., contained 7004 protein atoms, and surrounding water, altogether around 23,000 atoms. The model of CM, for the enzyme from *Bacillus subtilis*, contained 4192 protein atoms and 947 water molecules, in an approximate sphere of radius 25Å around the active site. To account for conformational variability of the enzymes, structures were taken from MM and QM/MM molecular dynamics simulations. (QM/MM molecular dynamics simulations, with semi-empirical QM methods, were also used to calculate activation free energies). These structures were used for reaction modelling, in which the structure is minimized at a series of set values of a reaction coordinate, defined in terms of breaking and forming bonds. Ten separate pathways were calculated for PHBH, and 16 for CM, with the results averaged over all paths. The paths were calculated at the B3LYP/MM level, which gives good structures.

Energies along the reaction pathways were calculated at the MP2, LMP2 and LCCSD(T0) levels (the L in the acronyms indicates that local approximations were used, and T0 is an approximate triples correction [27]), including the effects of the atomic point charges of the MM atoms in the calculations. These included by far the largest coupled cluster calculations ever performed (the calculations on PHBH involved 284 electrons and 1294 basis functions). Approximations included in the calculations were tested; for example, calculations were repeated with a much larger QM region for CM, showing a change in barrier of only 0.7 kcal/mol. The convergence with respect to basis set size was tested at the MP2 level, showing a change in barrier of less than 0.5 kcal/mol when very large basis sets were used. Similarly, tests showed the local approximations had an effect of less than 0.5 kcal/mol. Altogether, the errors in the best (LCCSD(T0)) barriers can be estimated to be less than 1 kcal/mol compared to extremely high (CCSD(T)) levels of quantum chemical theory, which are known, from calculations on small molecules, to give accurate barriers (i.e. typically within 1 kcal/mol of experiment. The accuracy obtained for energy barriers by Claeyssens *et al*. is unprecedented for enzyme reactions, and has been previously been difficult to attain even for small molecules.

How, then, do the calculated barriers compare with experimental findings for these enzymes? To make this comparison, the calculated (potential energy) barriers should be corrected for the quantum mechanical zero-point energy and thermal energy of molecular vibration: zero-point energy is calculated to reduce the barrier by 1.5 kcal/mol in CM, and by 1.1 kcal/mol in PHBH. In contrast, the thermal vibrational contribution is small (0.1–0.2 kcal/mol). With these corrections, the calculated barriers can be compared directly to experimental activation enthalpies. Experimental kinetic data for enzymes typically give steady-state rates, and so in making a comparison it is important to remember that a single chemical step may not be rate limiting – many enzymes have rates (at least partially) determined by conformational changes or product dissociation, for example. For both CM and PHBH, it has been suggested that other steps may be rate-limiting under some conditions. The chemical steps in both cases are thought to have barriers close to the apparent activation energy for the overall reaction.

The activation enthalpies calculated at the highest levels of quantum chemical theory (LCCSD(T0)) are in excellent agreement with experiment (within ~1 kcal/mol) for both enzymes. The calculated value for CM (average 13.3 kcal/mol, with a root mean square variation of 1.1 kcal/mol across 16 pathways) can be compared with the experimental value of 12.7 kcal/mol, while for PHBH the calculated and experimental activation enthalpies are 13.3 ± 1.5 kcal/mol and 12.0 kcal/mol, respectively. Only the LCCSD(T0) barriers are in close agreement with the experimental results. The LMP2 and B3LYP methods give barriers that are 3–5 kcal/mol too low. This shows that a high-level electron correlation treatment such as LCCSD(T0) is required for quantitative predictions of barrier heights (and probably other properties) in enzymes. In the transition state, more electrons are close together, and so the correlation energy is very different from that of the ground state. Electron correlation is therefore very important in determining the barrier.

The key quantity in determining reaction rates is the activation free energy (Δ^‡^*G*), not the enthalpy. To calculate free energy differences, the effects of protein motion should be included, i.e. averages over ensembles of structures. Activation free energies can be calculated from molecular simulations, such as molecular dynamics simulations. Such simulations are feasible for enzyme reactions with more approximate QM/MM methods, e.g. using semi-empirical QM techniques. In general, the best approach will be to calculate the free energy profile at the low level, and correct it based on the difference between low- and high-level QM/MM potential energy profiles. The difference between the average activation enthalpy and the activation free energy gives an estimate of the entropic contribution to the barrier. For both CM and PHBH, the estimated entropic contributions (at a temperature of 300 K) are small: 2.5 kcal/mol for CM (similar to the experimental value of 2.7 kcal/mol) and 0.4 kcal/mol for PHBH. Adding these entropic contributions to the calculated enthalpies at the highest QM/MM level gives free energy barriers very close to those obtained experiment: for CM, the (LCCSD(T0)) calculated Δ^‡^*G *is 15.6 ± 1.1 kcal/mol (versus 15.4 kcal/mol from experiment); for PHBH the calculated Δ^‡^*G *is 13.7 ± 1.5 kcal/mol, comparable to experimental values of 14–15 kcal/mol.

The agreement between calculated and experimental energy barriers is excellent for both enzymes. The comparison is made based on transition state theory, and the quality of the agreement indicates that transition state theory describes these enzyme-catalysed reactions well. Dynamical effects apparently play only a small role in determining the rate. Classical TST is known to be insufficient in some cases, but corrections for dynamical recrossing and quantum mechanical tunnelling can be included [2,17]. Despite some previous suggestions to the contrary, it seems that transition state theory provides a good general framework for understanding the rates of such enzyme-catalysed reactions.

Many challenges remain. Among these is the need for extensive conformational sampling to achieve convergence in free energies. In general, the best approach will be to calculate free energy profiles at a low level and correct them using high-level calculations. Determining reaction pathways can be difficult. Improvements to simple MM models (e.g. to include polarization and more sophisticated descriptions of electrostatics) are also likely to be necessary. The QM and MM methods should be consistent and balanced. The treatment of the QM region is only part of the challenge; it is important to have a good structural model of the surrounding enzyme (usually derived from X-ray crystallography), and to consider carefully, for example, the solvation of the protein and the protonation states of ionizable groups in the protein. The p*K*_a_s of basic and acidic amino acid sidechains can be significantly altered in the enzyme environment. Using the wrong protonation states could lead to the prediction of an incorrect mechanism. It is worthwhile to test methods of p*K*_a _prediction, particularly for active site residues. Predictions of amino acid p*K*_a_s in proteins can be made with simple but effective empirical methods such as PROPKA [28], or by finite-difference Poisson-Boltzmann calculations.

Given these many challenges, and the complexity of enzymes, validation of modelling methods by comparison with experiment will be very important. Many enzyme reactions involve several chemical and conformational changes [29,30], and the chemical step(s) may not always be rate limiting. Often experimental rates only for overall reaction under steady-state conditions are available. Experimental *k*_cat _rate constants do provide an upper limit on the barrier for any step in the mechanism, however. Even when the rate of a single step has been measured, this is likely to represent an average over many enzyme molecules in solution, whereas a calculated barrier is generally for a single molecule. Comparisons between experimental rates and calculated barriers should therefore be done with care. Transient kinetics and single molecule studies will be particularly useful for detailed comparisons. Detailed comparison with experiment will be a fascinating challenge, now that calculations based on first principles allow quantitative predictions on enzyme mechanisms to be made. These computational techniques also promise to make a significant contribution to other areas of biology and chemistry.

## Conclusion

The results of Claeyssens *et al*. show that electronic structure calculations can now predict activation barriers for enzyme-catalysed reactions with 'chemical accuracy', i.e. to within 1 kcal/mol in the best cases. At this level, quantitative, reliable predictions can be made about the mechanisms of enzyme-catalysed reactions. This development signals a new era of computational biochemistry.

## References

[B1] Mulholland AJ (2005). Modelling enzyme reaction mechanisms, specificity and catalysis. Drug Discovery Today.

[B2] Garcia-Viloca M, Gao J, Karplus M, Truhlar DG (2004). How enzymes work: Analysis by modern rate theory and computer simulations. Science.

[B3] Warshel A (2003). Computer simulations of enzyme catalysis: Methods, progress, and insights. Annu Rev Biophys Biomol Struct.

[B4] Martí S, Roca M, Andrés J, Moliner V, Silla E, Tuñón I, Bertrán J (2004). Theoretical insights in enzyme catalysis. Chem Soc Rev.

[B5] Claeyssens F, Harvey JN, Manby FR, Mata RA, Mulholland AJ, Ranaghan KE, Schütz M, Thiel S, Thiel W, Werner H-J (2006). High Accuracy Computation of Reaction Barriers in Enzymes. Angewandte Chemie Intl Edn.

[B6] Friesner RA, Guallar V (2005). Ab Initio Quantum Chemical and Mixed QuantumMechanics/Molecular Mechanics (QM/MM) Methods for Studying Enzymatic Catalysis. Ann Rev Phys Chem.

[B7] Himo F, Siegbahn PEM (2003). Quantum Chemical Studies of Radical-Containing Enzymes. Chem Rev.

[B8] Bathelt CM, Zurek J, Mulholland AJ, Harvey JN (2005). Electronic structure of Compound I in human isoforms of cytochrome P450 from QM/MM modelling. J Am Chem Soc.

[B9] Shaik S, Kumar D, de Visser SP, Altun A, Thiel W (2005). Theoretical perspective on the mechanism of cytochrome P450 enzymes. Chem Rev.

[B10] Bjelic S, Åqvist J (2004). Prediction of Structure, Substrate Binding Mode, Mechanism, and Rate for a Malaria Protease with a Novel Type of Active Site. Biochemistry.

[B11] Cleland WW, Frey PA, Gerlt JA (1998). The Low Barrier Hydrogen Bond in Enzymatic Catalysis. J Biol Chem.

[B12] Mulholland AJ, Lyne PD, Karplus M (2000). Ab initio QM/MM Study of the Citrate Synthase Mechanism. A Low-Barrier Hydrogen Bond is Not Involved. J Am Chem Soc.

[B13] Schutz CN, Warshel A (2004). The low barrier hydrogen bond (LBHB) proposal revisited: The case of the Asp … His pair in serine proteases. Proteins: Structure, Function, Bioinformatics.

[B14] Molina PA, Jensen JH (2003). A Predictive Model of Strong Hydrogen Bonding in Proteins: The Nδ1-H-Oδ1 Hydrogen Bond in Low-pH α-Chymotrypsin and α-Lytic Protease. J Phys Chem B.

[B15] Hur S, Bruice TC (2003). Just a Near Attack Conformer for Catalysis (Chorismate to Prephenate Rearrangements in Water, Antibody, Enzymes, and Their Mutants). J Am Chem Soc.

[B16] Strajbl M, Shurki A, Kato M, Warshel A (2003). Apparent NAC effect in chorismate mutase reflects electrostatic transition state stabilization. J Am Chem Soc.

[B17] Masgrau L, Roujeinikova A, Johannissen LO, Hothi P, Basran J, Ranaghan KE, Mulholland AJ, Sutcliffe MJ, Scrutton NS, Leys D (2006). Atomic Description of an Enzyme Reaction Dominated by Proton Tunneling. Science.

[B18] Lyne PD, Mulholland AJ, Richards WG (1995). Insights into Chorismate Mutase Catalysis from a Combined QM/MM Simulation of the Enzyme Reaction. J Am Chem Soc.

[B19] Senn HM, Thiel S, Thiel W (2005). Enzymatic Hydroxylation in p-Hydroxybenzoate Hydroxylase: A Case Study for QM/MM Molecular Dynamics. J Chem Theory Comp.

[B20] Guo H, Cui Q, Lipscomb WN, Karplus M (2001). Substrate conformational transitions in the active site of chorismate mutase: Their role in the catalytic mechanism. Proc Natl Acad Sci USA.

[B21] Guimarães CRW, Repasky MP, Chandrasekhar J, Tirado-Rives J, Jorgensen WL (2003). Contributions of Conformational Compression and Preferential Transition State Stabilization to the Rate Enhancement by Chorismate Mutase. J Am Chem Soc.

[B22] Claeyssens F, Ranaghan KE, Manby FR, Harvey JN, Mulholland AJ (2005). Multiple high-level QM/MM reaction paths demonstrate transition-state stabilization in chorismate mutase: correlation of barrier height with transition-state stabilization. Chem Commun.

[B23] Ridder L, Harvey JN, Rietjens IMCM, Vervoort J, Mulholland AJ (2003). Ab initio QM/MM modeling of the hydroxylation step in p-hydroxybenzoate hydroxylase. J Phys Chem B.

[B24] Ridder L, Mulholland AJ, Vervoort J, Rietjens IMCM (1998). Correlation of Calculated Activation Energies with Experimental Rate Constants for an Enzyme Catalyzed Aromatic Hydroxylation. J Am Chem Soc.

[B25] Wu T, Werner H-J, Manthe U (2004). First-Principles Theory for the H + CH_4_ —> H_2_ + CH_3_ Reaction. Science.

[B26] Manby FR, Werner H-J, Adler TB, May AJ (2006). Explicitly correlated local second-order perturbation theory with a frozen geminal correlation factor. J Chem Phys.

[B27] Schütz M (2000). Low-order scaling local electron correlation methods. III. Linear scaling local perturbative triples correction (T). J Chem Phys.

[B28] Li H, Robertson AD, Jensen JH (2005). Very Fast Empirical Prediction and Interpretation of Protein p*K*_a _Values. Proteins: Structure, Function, Bioinformatics.

[B29] Lodola A, Mor M, Zurek J, Tarzia G, Piomelli D, Harvey JN, Mulholland AJ (2007). Conformational effects in enzyme catalysis: Reaction via a high energy conformation in fatty acid amide hydrolase. Biophys J.

[B30] Pentikäinen U, Pentikäinen OT, Mulholland AJ (2007). Cooperative symmetric to asymmetric conformational transition of the apo-form of scavenger decapping enzyme revealed by simulations. Proteins: Structure, Function, Bioinformatics.

